# Synthetic Cathinones: Epidemiology, Toxicity, Potential for Abuse, and Current Public Health Perspective

**DOI:** 10.3390/brainsci14040334

**Published:** 2024-03-29

**Authors:** Shanshan Chen, Wenhua Zhou, Miaojun Lai

**Affiliations:** 1 Zhejiang Provincial Key Laboratory of Addiction Research, The Affiliated Kangning Hospital of Ningbo University, Ningbo 315201, China; chenshanshan3303@163.com (S.C.); zhouwenhua@nbu.edu.cn (W.Z.); 2 Department of Psychiatry, Ningbo Kangning Hospital, Ningbo 315201, China

**Keywords:** synthetic cathinones, neurotoxicity, abuse, adverse effects, new psychoactive substances

## Abstract

Synthetic cathinones, derived from cathinone found in the plant Catha edulis, represent the second largest and most frequently seized group of new psychoactive substances. They are considered as β-keto analogs of amphetamine, sharing pharmacological effects with amphetamine and cocaine. This review describes the neurotoxic properties of synthetic cathinones, encompassing their capacity to induce neuroinflammation, dysregulate neurotransmitter systems, and alter monoamine transporters and receptors. Additionally, it discusses the rewarding and abuse potential of synthetic cathinones drawing from findings obtained through various preclinical animal models, contextualized with other classical psychostimulants. The review also offers an overview of current abuse trends of synthetic cathinones on the illicit drug market, specifying the aspects covered, and underscores the risks they pose to public health. Finally, the review discusses public health initiatives and efforts to reduce the hazards of synthetic cathinones, including harm reduction methods, education, and current clinical management strategies.

## 1. Introduction

**Figure 1:**
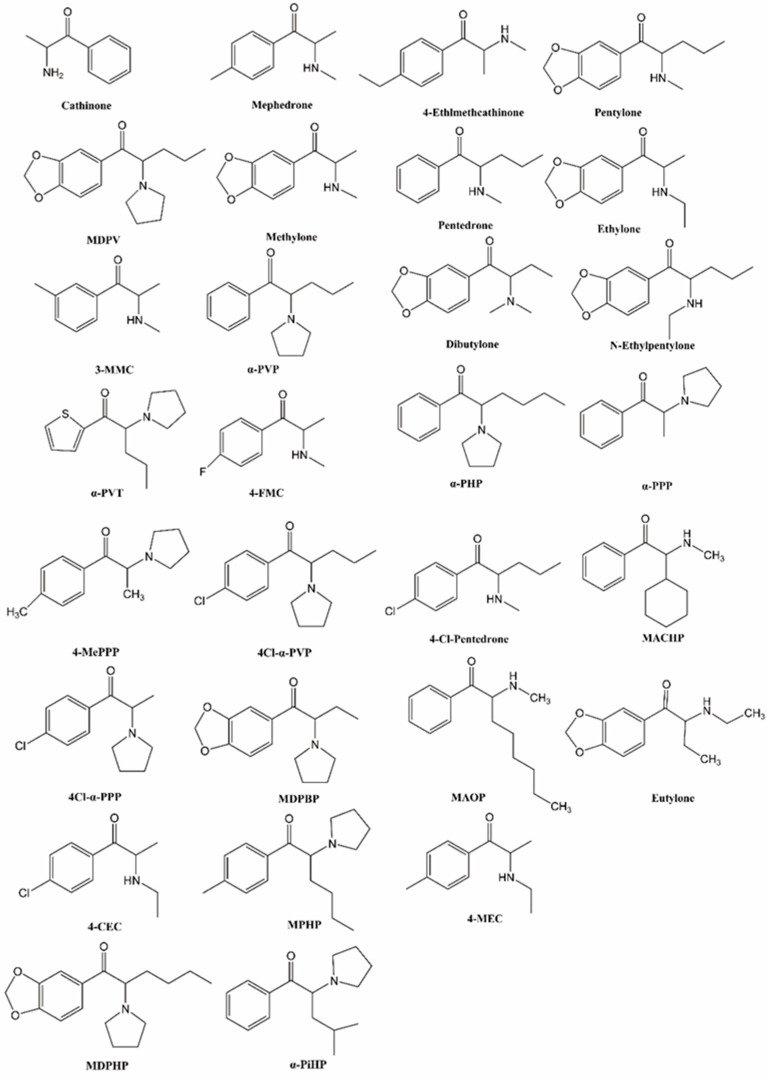
Chemical structures of cathinone and synthetic cathinones.

Cathinone (Figure 1), a β-keto analog of amphetamine, is a monoamine alkaloid found in Catha edulis (khat), producing psychostimulant effects akin to substances like amphetamine [1]. Synthetic cathinones (SCs) are β-keto analogues of cathinone, with cathinone’s backbone structure having four modification positions, the aromatic ring (R1), alkyl side chain (R2), and amino group (R3 and R4), allowing for the synthesis of a wide range of derivatives [2,3]. SCs can be classified into four sub-classes based on their structural modifications [3,4]: (1) N-alkyl cathinones, (2) N-pyrrolidine cathinones, (3) 3,4-methylenedioxy-N-pyrrolidine cathinones, and (4) 3,4-methylenedioxy-N-alkyl cathinones. Notably, 4-methylmethcathinone (mephedrone, Figure 1), 3,4-methylenedioxypyrovalerone (MDPV, Figure 1), and 3,4-methylenedioxy-N-methylcathinone (methylone, Figure 1) are the most commonly used SCs.

In the literature, some fundamental reviews about the development, pharmacokinetics, mechanisms of action, and biological/toxicological effects of synthetic cathinones can be found [5,6,7]. One of the main objectives of this review is to update the toxicity/neurotoxicity and abuse potential of SCs compared with other psychostimulants. Special focus is given to offer an overview of current trends of SCs in the illicit drug market, highlighting in particular the hazards of combining SCs with illegal drugs. In this context, we finally discuss public health initiatives and efforts to reduce the hazards of SCs, including harm reduction methods, education, and current clinical management strategies.

## 2. Materials and Methods

A literature search was performed to identify reported epidemiology, toxicity, potential for abuse, and the current public health perspective of SCs from 2000 to 2023. PubMed, Web of Science, and institutional and government websites were searched by two scientists for the following terms, alone or in combination: (“synthetic cathinone” OR “epidemiology” OR “toxicity” OR “neuroinflammation” OR “neurotransmitters” OR “mechanism” OR “abuse potential”) AND (“amphetamine” OR “METH” OR “MDMA” OR “toxicity” OR “neuroinflammation” OR “neurotransmitters” OR “mechanism”) AND (“new psychoactive substance” OR “synthetic cathinone” OR “amphetamine” OR “combination” OR “public health perspective”).

The selected publications’ referenced articles were also considered. Articles were screened according to the following criteria:

1. English or Chinese language.
2. Studies involving human or animal exposure to SCs, alone or in combination with other drugs from 2000 to 2023.
3. Exposure confirmation through qualitative or quantitative toxicology analyses of human biological matrices.

All the data were extracted, curated, and analyzed without software aid. The articles were manually screened by three of the authors and a second check was performed to avoid researcher bias.

## 3. Results

### 3.1. History and Epidemiology

In the late 1920s, methcathinone and mephedrone, two synthetic derivatives of cathinone, were synthesized for medicinal purposes, leading to the development of numerous other molecules [8]. Initially, SCs were developed as antidepressants and appetite suppressants, but their potential for abuse and dependence limited their therapeutic use [9,10,11]. Over time, SCs gained popularity as “legal highs” and started appearing in European markets through online platforms in the early 2000s [12]. They were commonly sold under names like “plant food”, “bath salts”, or “research chemicals”, usually in the form of crystals, white powder, or capsules [13]. Due to their stimulant effects, easy availability, and former legal status, SCs quickly became widely used worldwide [14].

The popularity of SCs had severe consequences, with reports of their misuse, particularly with mephedrone in 2009–2010, being associated with toxicity and deaths [15,16,17]. In response, mephedrone was classified as a class B drug under the UK Misuse of Drugs Act 1971 in 2010, and the Council of the European Union decided to control it in European countries [18,19]. However, this did not stop the emergence of other substances. In fact, 26 new derivatives replaced mephedrone, which were first reported in the EU Early Warning System [20,21,22]. Between 2012 and 2015, the market saw a staggering influx of 69 new derivatives, reaching a peak of 31 new derivatives in 2014 alone [23,24,25,26]. Over the past decade, the illicit drug market has undergone significant transformations, marked with the introduction of new and previously unknown psychoactive substances annually. Despite the implementation of regulatory measures and early warning systems, NPSs (new psychoactive substances), including SCs, continue to be easily accessible and widely utilized [27]. Following a rapid expansion from 2009 to 2018, the number of NPSs available on the market has stabilized at approximately 550 per year. In 2020, 548 NPSs were reported, with 77 identified for the first time. However, this figure decreased to 50 in 2021. According to the World Drug Report 2023, the cumulative number of NPSs reached 1165 substances in 2021 and is anticipated to reach 1184 substances in 2022 [28]. SCs, as the second largest and second most frequently seized group of NPSs, have generally maintained stable numbers, with some reported declines for 2020. In 2021, the UNODC reported a total of 201 synthetic cathinone substances [29]. Furthermore, the quantity of seized SCs in 2020 was 98 percent lower than its peak in 2015. SCs have shown a significant prevalence in specific marginalized rural populations in Hungary, where polysubstance use is prevalent. Nevertheless, there has been an overall decrease in the use of NPSs in the United States and certain Western and Central European countries, following an initial surge in usage.

The Commission on Narcotic Drugs, mandated by three International Drug Control Conventions, holds authority to assess and determine the global control and scheduling of substances. Currently, there are a total of 17 synthetic cathinones scheduled worldwide, with the majority added since 2015 [30]. Despite these efforts, drug designers consistently outpace law enforcement, leading to a continual influx of new derivatives in the drug market, often serving as substitutes for previously illicit substances. While regulatory controls on SCs exist in many countries [30], the continuous development of novel synthetic cathinone analogues through chemical structure modifications remains a significant public health challenge.

### 3.2. Toxicity

SCs are commonly categorized based on their pharmacological action, properties, or in comparison to traditional stimulant drugs. A range of SCs, including mephedrone, methylone, and ethylone, produce effects similar to those of cocaine and 3,4-methylenedioxymethapmphetamine (MDMA). These substances act as substrates for noradrenaline (NA), dopamine (DA), and serotonin (5-HT) transporters (NET, DAT, and SERT, respectively) [31]. Another category of SCs, including methcathinone, ethcathinone, and flephedrone, serve as monoamine transporter substrates with high inhibitory potencies at DAT and lower inhibitory potencies at SERT. They also induce the release of NE and DA in a manner similar to methamphetamine (METH) [31]. Mephedrone exhibits a higher inhibitory potency at SERT compared to its DAT activity, while also promoting the release of both NE and 5-HT in the striatal region of the nucleus accumbens, similar to amphetamine analogues such as MDMA and paramethoxymethamphetamine [32,33].

The significant differences in the potency and affinity of SCs at the monoamine membrane transporters and receptors result in specific clinical and toxic effects. For example, dopaminergic effects can lead to psychostimulant effects and reinforcing properties; noradrenergic effects can cause sympathomimetic stimulation, and serotonergic effects can lead to hyperthermia, seizures, paranoia, and hallucinations [31]. Indeed, the toxicological profile of SCs is mainly related to their pharmacological action rather than to their chemical structures.

SCs are commonly taken orally as capsules or tablets, as well as nasally by dipping a key into powder and inhaling it. They can also be administered through intravenous or intramuscular injections, and, in some cases, used as enemas [34]. Due to their high-water solubility, the way SCs are administered can affect their pharmacokinetics. The typical single oral dose of SCs ranges from 25 to 75 mg, with low doses usually ranging from 5 to 15 mg, while high doses are typically above 90 mg. After oral administration, SCs demonstrate a rapid onset of action within a few minutes, reaching peak concentration at approximately 30 min, followed by a rapid decrease in concentration [35].

SCs are often abused for the purpose of enhancing energy levels and sensory experiences. Abusing these substances can result in various side effects, including euphoria, high energy, mania, and other symptoms in the early stages. In later stages, individuals may experience drowsiness, nosebleeds, delusions, anxiety, hallucinations, insomnia, sweating, nausea, vomiting, and general pain. Prolonged abuse of SCs can result in addiction, as well as a range of physical and psychological damage, including the possibility of fatalities [36].

Toxicological studies have employed various benchmarks to evaluate SC-induced neurotoxicity, including inflammation, the disruption of monoaminergic properties of monoaminergic neurotransmitters, and their transporters and receptors. The toxicity of amphetamine includes prominent neurological and cardiovascular effects. In the case of an amphetamine overdose, patients may experience nystagmus, agitation, confusion, delusions, and seizures [37,38]. METH use can lead to a range of cardiovascular issues including hypertension, arrhythmias, coronary vasospasm, myocardial infarction, and cardiomyopathy [39]. In comparison to substances like METH and MDMA, SCs generally exhibit a lower level of neurotoxicity. However, SCs pose a higher risk of overdose compared to their non-beta-ketone relatives. Overdose cases involving SCs often initially present signs of increased agitation, violent behavior, aggression, and psychosis [40], and can progress to multiple organ failure, similar to other psychostimulant drugs [41]. Renal symptoms, such as elevated blood urea nitrogen levels, dehydration, and hyponatremia, may indicate peripheral toxicity as a major cause of death from cathinone. Cardiac injury, characterized by a reduced cardiac output, sinus tachycardia, and cardiac arrest, has been observed with SCs such as MDPV [42], methylone [43], 3-methylmethcathinone (3-MMC, Figure 1) [44], and mephedrone [16]. Additionally, SC overdose cases have been associated with hepatoxicity, indicated by elevated levels of aspartate and alanine aminotransferase [45], and, specifically, hepatic portal and lobular inflammation in methylone overdose victims [46]. It is challenging to determine the precise risks of individual cathinones based solely on available data due to the use of combinations of SCs with each other and/or various other illicit drugs.

#### 3.2.1. Neuroinflammation

The main neurotoxicity of non-keto amphetamines is the ability to trigger inflammatory processes in terminally degenerated brain regions [47]. Amphetamine neurotoxicity involves the inhibition of biosynthetic enzymes responsible for monoamine production and the inactivation of tyrosine hydroxylase. Additionally, the use of amphetamines can lead to a decrease in the functioning of vesicular monoamine transporter-2 (VMAT2). In unmyelinated axons at nerve endings, it can also cause degeneration and apoptosis [48]. Research has shown that chronic neuroinflammation results in elevated levels of cytokines derived from glial cells. These cytokines exert neurotoxic effects on vulnerable neurons, thereby implicating glial activation as a contributing factor in the events leading to neuronal damage [49].

Neurotoxicity studies on SCs primarily focus on the toxicity of mephedrone. However, the exact mechanisms underlying mephedrone-induced neurotoxicity have not been fully elucidated. Despite this, there is overwhelming evidence of its potential danger. Interestingly, mephedrone does not cause DA neurotoxicity, but instead amplifies the neurotoxic effects of METH, amphetamine, and MDMA on these nerve endings [50]. In adolescent rats, mephedrone was founded to reduce the densities of DAT and SERT in the frontal cortex. Importantly, this effect did not involve microgliosis, an inflammatory response in brain tissue [51]. In vivo studies have indicated that the administration of mephedrone did not activate astroglia or microglial cells in the striatum [52]. However, Martinez-Clemente reported that mephedrone induced a dose- and time-dependent neurotoxicity in both dopaminergic and serotoninergic systems in mice [53]. Marszalek-Grabska reported that a binge-like mephedrone treatment resulted in memory deficits and a significant reduction in kynurenic acid levels in the brains of mice. Additionally, in vitro studies demonstrated that mephedrone caused a minor decline in cell viability and proliferation [54]. Studies showed that rats who received mephedrone in adolescence displayed deficits in spatial memory and reversal learning during adulthood. These effects were found to be associated with alterations in the level of matrix metalloproteinase-9 [55]. In addition to its effects on brain tissue, mephedrone has been shown to induce oxidative stress in the hearts, kidneys, spleens, and livers of mice [56].

The neurotoxicity of various SCs has also been investigated. For instance, the self-administration of α-pyrrolidinopentiophenone (α-PVP, Figure 1) or mephedrone in male rats has been shown to elevate levels of inflammatory cytokines, including interleukin-1 alpha (IL-1α), IL-1 beta, IL-6, and tumor necrosis factor-alpha (TNF-α) in the brain. Conversely, the administration of SCs was more likely to increase the levels of inflammatory cytokines in the plasma of female rats [57]. A dose of 20 mg/kg of α-PVP was observed to significantly impact affect spatial learning and memory, as well as the brain mitochondrial protein yield and mitochondrial function. In contrast, a lower dose of α-PVP (5 mg/kg) did not produce any noticeable effects on spatial learning and memory or brain mitochondrial function [58]. The repeated administration of alpha-pyrrolidinopentiothiophenone (α-PVT, Figure 1) may activate Toll-like receptor 4 (TLR4), leading to neuroinflammation through TLR-mediated NF-κB and MAPK signaling pathways. This activation may result in the production of TNF-α and IL-6 in the striatum in mice [59]. Methylone and MDPV were found to decrease cell viability in both differentiated and undifferentiated DA cells in a dose-dependent manner [60]. When combined with each other or with mephedrone, these substances did not result in changes in glial fibrillary acidic protein (GFAP) levels in the striatum of mice. An acute MDPV binge failed to cause striatal dopaminergic terminal damage and to alter glial activity [61]. However, a repeated binge-like intake of MDPV causes changes in cytokine levels in the prefrontal cortex that persist into the abstinence period [62]. Methylone can enhance the expression of GFAP induced by METH by approximately 50% [63].

#### 3.2.2. Neurotransmitters

METH, amphetamine, and MDMA are known to induce neurotoxic effects on monoaminergic systems, partly attributed to alterations in DA and 5-HT transporters and receptors [64]. METH increases the release of both DA and 5-HT by directly and indirectly affecting DAT and SERT, resulting in significant toxicity to DA nerve terminals in the striatum [65]. Amphetamine can disrupt the function of VMAT-2 and the vesicle proton gradient, leading to an increase in cytoplasmic levels of DA and 5-HT by releasing them from vesicular compartments [66]. Similarly, MDMA increases 5-HT release and exhibits a stronger affinity for SERT than DAT [67].

Similar to METH and amphetamine, methcathinone exhibits high inhibitory potency at DAT and low potency at SERT [68]. The repeated administration of methcathinone in animals induced significant decreases in the levels of DA and 5-HT in the striatum, as well as the activity of the serotoninergic enzyme [69]. Methcathinone users also display a reduction in DAT density [70]. The deficits in DAT and SERT induced by methcathinone are believed to contribute to the persistence of damage to the DA and 5-HT systems.

In mice, it has been discovered that mephedrone reduces the quantity of D_2_ receptors in the striatum, as well as the quantity of 5-HT_2A_ receptors in the prefrontal cortex and hippocampus. Mephedrone induces long-term damage to the dopaminergic and serotoninergic systems in mice, resulting in the loss of DAT and SERT [53]. However, following exposure to mephedrone, there is an increase in D_3_ receptors in the striatum [71]. In terms of selectivity ratios, mephedrone has DAT/SERT ratios and NET/DAT ratios close to unity, comparable to those seen with MDMA [72].

Methylone is also known to inhibit NET and DAT but is slightly less potent against SERT. Methylone exhibits selectivity profiles similar to mephedrone, but is approximately half as potent. Similar to mephedrone, methylone acts as a partial agonist at 5-HT_1A_ receptors with low potency and weekly antagonizes 5-HT_2A_ receptors.

MDPV has a very high affinity for both DAT and NET, being at least ten times more potent for DAT than cocaine and METH [68]. However, MDPV has a weak inhibitory effect on SERT, with DAT/SERT inhibition ratios greater than 100. Unlike mephedrone, MDPV exerts high potency in the inhibition of both NET and DAT, while exhibiting a slight activation of 5-HT receptors, leading to high DAT/SERT inhibition ratios.

The potency of SCs that inhibit monoamine transporters may be related to their chemical structures. Methcathinone with para-(4) substitution, including mephedrone, 4-fluoromethcathinone (4-FMC, Figure 1), and 4-ethylmethcathinone, had relatively more serotonergic neurotoxicity compared with methcathinone [73]. The carbonyl and extended alpha-alkyl groups in MDPV contributed more significantly to the drug’s affinity for DAT than the methylenedioxy group. Research on N-ethyl-hexedrone analogues has demonstrated that the potency of DA uptake inhibitors increases as the aliphatic side chain extends from methyl to propyl. However, as the chain length increases from butyl to pentyl, the potency decreases [74]. On the other hand, longer α-carbon side chains of SCs resulted in increased cytotoxic properties in PC12 cells, probably due to their enhanced membrane penetration [74].

### 3.3. Abuse Potential

As mentioned above, SCs share common pharmacological effects with amphetamine, leading to an increased release of monoamines such as DA, NE, and 5-HT. These neurotransmitters are believed to play a role in the euphoric and rewarding effects of various drugs of abuse. The fact that SCs act on reward-related neurotransmitter substrates and are self-administered by humans suggests that they have the potential for abuse [75]. Due to the limitations associated with human experiments and the wide variety of SC compounds present on the illicit drug market, it becomes imperative to depend on preclinical animal behavioral models for a more comprehensive understanding of their abuse potential. The three predominant animal models commonly employed for evaluating substance abuse potential include self-administration, discriminative stimulation, and conditioned place preference.

#### 3.3.1. Self-Administration and Self-Stimulation

The intravenous self-administration (IVSA) model in animals is widely recognized for its face validity for simulating human drug administration behavior. This model allows for the assessment of reinforcing effects through various procedures. Dose–response analyses are used to determine the specificity and reinforcing potency. Drug substitution assessments are used to compare similarities and differences between drugs. The progressive ratio schedule or behavioral economic procedure are utilized to evaluate the relative reinforcing efficacy of SCs compared to other illicit drugs, as differences in reinforcing potency among drugs may not necessarily predict their relative reinforcing efficacy [76,77,78]. Additionally, they facilitate the investigation of the reinstatement of drug-taking behavior induced by drugs, stress, and drug-associated cues after self-administration [79].

The selectivity and sensitivity of intracranial self-stimulation (ICSS) in identifying the potential of drug abuse are comparable to those of IVSA procedures [80]. In ICSS experiments, the manipulation of frequency or amplitude of stimulation is employed to elicit probabilities or a wide range of baseline response rates. The well-established ability of several SCs to influence the brain-stimulation-rewarded (BSR) thresholds for ICSS further underscores their potential for abuse.

Initial studies primarily focused on investigating the reinforcing effects of cathinone and its first-generation synthetic derivatives compared to illicit drugs. The confirmation of abuse liability of these substances stems from reliable self-administration observed in animal models. Early assessments of mephedrone revealed that rats readily acquired responses to it, displaying higher response rates to the same dose of METH [81]. Progressive ratio assessments and dose–response substitution demonstrated that mephedrone exhibits an equal reinforcing efficacy to METH and effectively substitutes for METH in METH self-administered animals [82]. Notably, mephedrone has a greater reinforcing efficacy than methylone, leading to overall higher response rates in female Sprague Dawley rats [83] and male Wistar rats [82]. Research exploring the impact of mephedrone on ICSS reward thresholds in mice observed a dose-dependent reduction in BSR thresholds, indicating a high potential for abuse [84]. Watterson et al. were the first to demonstrate methylone’s dose-dependent reinforcing properties in male rats, suggesting a potential for addiction comparable to or even greater than MDMA [85].

The initial evaluation of IVSA of MDPV demonstrated its support for self-administration to a similar degree as METH. In dose–response and progressive ratio assessments, MDPV induced higher levels of responding compared to METH, indicating its stronger reinforcing properties [86]. However, a study by Watterson et al. reported that MDPV and METH produced a comparable level of responding when administered at a dose of 0.05 mg/kg/infusion in progressive ratio assessments. This suggested that at this dosage, the reinforcing effects of MDPV and METH were similar [87]. Schindler et al. conducted a study showing that MDPV exhibited higher efficacy compared to cocaine, and both MDPV and cocaine as being more potent than methylone in terms of their reinforcing effects [88]. Subsequent assessments confirmed that MDPV has a higher effectiveness and potency compared to cocaine and METH [89]. Additionally, in the ICSS experiments, methcathinone, MDPV, methylone, and mephedrone were found to induce the facilitation of ICSS in a dose- and time-dependent manner, with the order of effects being methcathinone ≥ MDPV ≥ methylone > mephedrone [90]. MDPV specifically led to reductions in ICSS thresholds, whereas methylone only exhibited trends towards this effect [85,87].

Following the initial investigations, the second generation of SCs underwent scrutiny, including compounds such as α-PVP, α-PVT, α-pyrrolidinohexiophenone (α-PHP, Figure 1), and α-pyrrolidinopropiophenone (α-PPP, Figure 1), and others. Notably, α-PVP has been the subject of extensive study. Recent studies indicates that α-PVP exhibits comparable potency and efficacy to MDPV in terms of reinforcing effects [91]. Moreover, both the racemate and the S and R enantiomers of α-PVP induce self-administration in a dose-dependent manner, with the order of potency being S enantiomer > racemate >> R enantiomer [92]. Compounds such as α-PVP, α-PHP, and α-PPP have been shown to increase spontaneous activity and decrease brain reward thresholds in a dose-dependent manner in female rats [93]. In a study by Huskinson et al., a behavioral economics evaluation was conducted to assess the reinforcing efficacy of α-PVP and 4-MePPP in comparison to METH. The results indicated that both α-PVP and 4-MePPP were more effective reinforcers than METH [77]. Experiments conducted in rhesus monkeys, designed to examine demand elasticity, revealed the rank order of reinforcing efficacy as follows: cocaine > MCAT = methylone > α-PVP = MDMA [94]. Xu et al. demonstrated that the reinforcing potency of α-PVP surpassed that of 4-chloro-α-pyrrolidinopentiophenone (4cl-α-PVP, Figure 1), 4-chloro-α-pyrrolidinopropiophenone (4cl-α-PPP, Figure 1), and METH [78]. Additionally, Cheong et al. reported that α-PVT exhibited self-administration in rats, and an inverted U-shaped dose–response curve was observed [95]. Accumulating evidence has shown that pentylone and pentedrone have a higher reinforcing potency and efficacy compared to methylone [96]. In a study by Lai et al., the abuse potential of ethylone, dibutylone, and N-ethylpentylone was compared with METH using a fix ratio self-administration schedule. Results showed all three cathinones acted as reinforcers, but their reinforcing potency were lower than that of METH; however, the rank order of reinforcing effectiveness was determined to be METH ≈ dibutylone > N-ethylpentylone ≈ ethylone, based on the demand elasticity of the economic demand curve [97]. The reinforcing potency and efficacy of SCs compared with each other or with other psychoactive drugs are summarized in Table 1.

#### 3.3.2. Drug Discrimination Learning

Drug discrimination is a research method employed to compare the subjective effects of a compound with other drugs known for their abuse potential. This procedure has revealed that methylone, mephedrone, and MDPV can fully substitute for the discriminative stimulus effects of both cocaine and METH, indicating shared interoceptive effects [98,99,100]. Conversely, METH and MDMA also substituted for the effects of MDPV, but they had decreased response rates at varying doses [101]. Similarly, under comparable training conditions, α-PBP, α-PVP, α-PHP, α-PPP, 4cl-α-PVP, 4cl-α-PPP, and ethylone demonstrated dose-dependent substitution for the discriminative stimulus effects of both cocaine and METH. On the other hand, 4′-MePPP, α-PVT, and 3′, 4′-Methylenedioxy-alpha-pyrrolidinobutyrophenone (MDPBP, Figure 1) exclusively replicated the discriminative stimulus effects of METH [78,102,103]. Dipentylone, N-ethylhexedrone, 4-chloroethcathinone (4-CEC, Figure 1), and 4-methyl-α-phrrolidinohexiophenone (MPHP, Figure 1) fully substituted for the discriminative stimulus effects of METH and cocaine, although only 4-CEC fully substituted for MDMA [104]. In a study by Shetty et al., the drug discrimination effects of 3,4-methylenedioxy-alpha-pyrrolidinohexanophenone (MDPHP, Figure 1), 4-Cl-α-PPP, alpha-pyrrolidinoisohexiophenone (α-PiHP, Figure 1), and 4-chloro-pentedrone (4-Cl-pentedrone, Figure 1) were compared to METH and cocaine. They found that all test compounds fully substituted for the discriminative stimulus effects of cocaine, but only 3,4-MD-α-PHP, α-PiHP, or 4-Cl-α-PPP fully substituted for the discriminative stimulus effects of METH [105].

Drug discrimination studies have expanded their focus to explore the discriminative stimulus effects of drug mixtures, particularly in the context of “bath salt” products that are often intentionally mixed or adulterated with another compound. Collins et al. examined the discriminative stimulus effects of cocaine, caffeine, and MDPV, both individually and in binary mixtures (specifically, cocaine/caffeine, caffeine/MDPV, and MDPV/cocaine at fixed-dose ratios of 3:1, 1:1, and 3:1). The findings revealed that METH and MDPV had a higher potency compared to cocaine and caffeine in inducing dose-dependent cocaine-appropriate responding. Additionally, the binary mixtures generally exhibited additive effects [106]. In squirrel monkeys, Alison et al. found that MDPV, α-PVP, and MCAT fully substituted for METH, but only partially substituted for MDMA. In contrast, mephedrone and methylone fully substituted for MDMA, but failed to fully substitute for METH [107].

#### 3.3.3. Conditioned Place Preference

The conditioned place preference (CPP) model is a widely used paradigm for studying the rewarding effects of drugs and evaluating the abuse potential of new psychoactive substances. Lisek et al. were the pioneers in employing the CPP model to assess the rewarding effects of SCs. They reported that rats injected with 30 mg/kg of mephedrone demonstrated a significant preference for the drug-associated chamber compared to the saline group [108]. Likewise, mice that were conditioned with mephedrone also displayed a significant place preference in the CPP model [109]. Wronikowska et al. confirmed that mephedrone produced rewarding effects in the CPP paradigm and further revealed that memantine could reverse the expression of this effect [110]. In a study comparing the rewarding effects of mephedrone, MDPV, and methylone using the CPP model in mice, Kaelsson et al. demonstrated that all compounds produced CPP in a dose-dependent manner. Notably, MDPV exhibited a higher potential for CPP compared to mephedrone and methylone [111]. Additionally, several other SCs have been reported to induce CPP, including α-PVP [112], α-PVT [95], α-PHP [103], and 4-MePPP [103]. These drugs could induce CPP, indicating their potential for addiction, although it does not definitively indicate their ability to support self-administration. For instance, both 2-cyclohexyl-2-(methylamino)-1-phenylethanone (MACHP, Figure 1) and 2-(methylamino)-1-phenyloctan-1-one (MAOP, Figure 1) produced CPP, but only MACHP was found to be self-administered [113]. Eutylone, one of the third-generation SCs, produced dose-dependent CPP in male mice [114] and female rats [115]. Interestingly, pre-exposure to cocaine and MDMA did not have any effects on the development of eutylone-induced CPP [114].

## 4. Current Public Health Perspective

According to the World Drug Report 2023 [28], qualitative assessments suggested an increase in the use of amphetamines in 2021 and over the last decade, with an estimated 36 million people using amphetamines and an estimated 20 million people using “ecstasy”-type substances in 2021. METH manufacture and use have spread beyond the traditional markets for the drug, namely, East and Southeast Asia and North America, most notable into the southwest, Europe, and Africa. The use of NPSs may be decreasing in North America and Europe, but Eastern Europe, Asian, and Africa are likely experiencing mid-term increases in use. A total of 44 countries reported seizures of synthetic NPSs most commonly involving ketamine, followed by SCs and cannabinoids in 2020 and 2021. A survey conducted in the European region involved nearly 100,000 high school students aged 15–16 years old, revealing a stable or slightly decreasing trend in NPS use. The 2019 survey found that the average prevalence of NPS use in Europe was nearly equal for boys and girls across the 23 participating countries. Approximately 3.4% of boys and 3.3% of girls reported having used NPSs at least once in their lifetime [116]. However, the European Drug Report 2023 indicates a diversification of the drug market in the European region. Furthermore, there are signs that METH and SCs are exerting a more substantial impact on stimulant-related issues in Europe than in the previous year [117].

Compared to plant-based drugs, synthetic drugs offer criminal actors a means of reducing risk and operational costs. These drugs can be manufactured with higher purity owing to advancements in synthesis and refinement processes. Additionally, synthetic compounds often exhibit much higher potency than their naturally occurring counterparts. The actual composition of NPSs sold online may vary significantly from the package label, leading to potential confusion for consumers. Consumers often perceive various stimulants as interchangeable and may be inclined to experimenting with new products based on their availability on the market. This trend raises concerns, as synthetic stimulants often share a similar appearance, whether in the form of powders or tablets. This similarity makes it challenging for consumers to discern the specific substance or mixture they are consuming. The potential consequences of engaging in such high-risk behaviors include exposure to more potent and unfamiliar substances, leading to adverse health outcomes such as acute and chronic mental health issues, intoxication, infectious disease, and even fatalities. It is crucial to raise awareness about these risks and implement preventive measures to safeguard public health. It has been shown that SCs may be adulterated with each other or with other illegal drugs such as ecstasy or MDMA [118,119,120,121,122,123]. Due to the similar effects produced by SCs and amphetamines, users may not notice the adulteration or replacement of amphetamines with SCs, and, thus, the risk of overdose and death can be greatly increased. One of the first reported cases of death from unintentional SC overdose involved a female who died after consuming two pills, which turned out to be high doses of methylone and butylone instead of ecstasy [124]. From 2017 to 2020, a total of 31 different SCs were identified in 75 documented fatal intoxication cases reported in the literature, alone or in combination with other substances [125]. On the other hand, unintentional use of SCs carries a significant risk of death from self-harm. A 3-year review of NPSs in casework revealed that among fatalities following SC use, 41% were hangings or other forms of mechanical suicide (i.e., fatal self-harm), representing the highest proportion compared to other classes of drugs [126]. A number of preclinical studies have begun to elucidate the neurotoxic and behavioral effects of drug combinations. Rats pretreated with mephedrone and cocaine mixtures exhibited an augmented response to cocaine following drug abstinence [127]. An MDPV/mephedrone combination resulted in enhanced stimulant effects in the rats [128]. Mixtures of MDPV and METH produced higher increases in locomotor activity compared to either drug alone [129]. Combining methylone with caffeine resulted in an enhanced reinforcing effectiveness compared to methylone alone [130]. When cannabidiol was administered concurrently with MDPV during self-administration, it resulted in an increase in drug-seeking and taking behaviors; albeit, this effect was observed only in the high-responder group of mice. Furthermore, cannabidiol demonstrated anxiolytic-like effects specifically in MDPV-treated mice [131].

Drug use disorders and other mental health problems are intricately linked: mental health problems heighten the risk of developing drug use disorders, while drugs can aggravate existing mental health problems if used without medical supervision. Therefore, prioritizing the management of mental health concerns in drug prevention and treatment has become increasingly imperative. To reduce the risk of escalating drug use disorders, especially given the high prevalence of mental health conditions, it is imperative to implement large-scale prevention initiatives that involve schools, families, and communities. These comprehensive programs aim to educate individuals about the dangers of drug use, particularly the use of SCs in combination with other psychoactive drugs, to provide necessary support services and to cultivate a supportive environment. In the clinical treatment of SCs, clinicians should recognize the typical symptoms of SC overdose, such as agitation, sustained hyperthermia, and psychosis. The standard treatment protocol involves promote cooling, hydration, and the administration of antipsychotic medications. It is crucial for both drug users and clinicians to be mindful of the risk of self-harm and suicide among patients during intoxication and withdrawal. Typically, the management of SCs and other NPSs or unknown psychotropic ingestion focuses on addressing the adverse effects that may arise [132]. Due to the similarity of SCs with other stimulants, symptom-directed supportive care similar to those recommended for intoxication with those drugs might be useful. In general, the use of atypical antipsychotics, including olanzapine, has shown good efficacy in containing episodes of aggression [133]. Finally, treatment for patients with prolonged exposure to SCs should ideally include a drug management plan coupled with psychotherapy [134].

## 5. Conclusions

The abuse of SCs is still a serious public health concern, and the emergence of novel derivatives with unknown chemical and biological properties raises significant challenges for law enforcement, healthcare providers, and researchers. The present review summarized the toxicity/neurotoxicity and abuse potential of SCs compared with other psychostimulants. Although the neurotoxicity induced by SCs, including inflammation, oxidative stress, and cytotoxicity, appears to be more moderate compared to amphetamines, the potential for abuse of SC potential is high. The review also offered an overview of current trends of SCs on the illicit drug market, highlighting, in particular, the hazards of the combination of SCs and illegal drugs. However, a better understanding of synergistic effects of SCs on toxicity and abuse potential in combination with other psychostimulants would be important for future efforts aimed at limiting the societal impact of mixed use of these drugs in reality, as well as for the prevention and treatment of their toxicological effects and potential overdose.

## Figures and Tables

**Table 1:** The reinforcing potency and efficacy of synthetic cathinones compared with each other or with other psychoactive drugs.

Synthetic Cathinones	The Reinforcing Potency	The Reinforcing Efficacy
Mephedrone	≥METH [81,82]	=METH [82]
Methylone	<mephedrone [82,83], >mephedrone [90]	<cocaine [88]
	>MDMA [85]	
	<cocaine [88]	
MDPV	>METH [86,89]	≥METH [87,89]
	≥methylone [88]	>cocaine [88,89]
	>cocaine [89]	
α-PVP	=MDPV [91],	=MDPV [91], <Methylone [94]
	=4-MePPP [77]	>METH [77]
	>METH >4cl-α-PVP >4cl-α-PPP [78]	=MDMA [94]
		<cocaine [94]
Pentylone, pentedrone	>methylone [96]	>methylone [96]
Ethylone, dibutylone, N-ethylpentylone	<METH [97]	<METH [97]

## Abbreviations

SCs	synthetic cathinones
Mephedrone	4-methylmethcathinone
MDPV	3,4-methylenedioxypyrovalerone
Methylone	3,4-methylenedioxy-N-methylcathinone
CNS	central nervous system
METH	methamphetamine
MDMA	3,4-methylenedioxymethapmphetamine
3-MMC	3-methylmethcathinone
NA	noradrenaline
DA	dopamine
5-HT	serotonin
NET	noradrenaline transporter
DAT	dopamine transporter
SERT	serotonin transporter
VMAT2	vesicular monoamine transporter 2
α-PVP	α-pyrrolidinopentiophenone
IL-1α	interleukin-1 alpha
TNF-α	tumor necrosis factor-alpha
α-PVT	alpha-pyrrolidinopentiothiophenone
TLR4	Toll-like receptor 4
GFAP	glial fibrillary acidic protein
4-FMC	4-fluoromethcathinone
IVSA	intravenous self-administration
ICSS	intracranial self-stimulation
BSR	brain stimulation reward
α-PHP	α-pyrrolidinohexiophenone
α-PPP	α-pyrrolidinopropiophenone
4-MePPP	4-methyl-alpha-pyrrolidinopropiophenone
4cl-α-PVP	4-chloro-α-pyrrolidinopentiophenone
4cl-α-PPP	4-chloro-α-pyrrolidinopropiophenone
MDPBP	3,4-Methylenedioxy-alpha-pyrrolidinobutyrophenone
4-CEC	4-chloroethcathinone
MPHP	4-methyl-α-phrrolidinohexiophenone
MDPHP	3,4-methylenedioxy-alpha-pyrrolidinohexanophenone
α-PiHP	alpha-pyrrolidinoisohexiophenone
4-Cl-pentedrone	4-chloro-pentedrone
CPP	conditioned place preference
MACHP	2-cyclohexyl-2-(methylamino)-1-phenylethanone
MAOP	2-(methylamino)-1-phenyloctan-1-one
MSM	men who have sex with men
GHB	gamma-hydroxybutyrate
GBL	gamma-butyrolactone
SDU	sexualized drug use
4-MEC	4-methylethcathinone
NPSs	new psychoactive substances
